# Extending infection prevention and control nursing (IPCN) provision to weekends at one National Health Service hospital in the United Kingdom: A feasibility and acceptability study

**DOI:** 10.1177/17571774231165406

**Published:** 2023-03-23

**Authors:** Valerie Brueton, Lorraine Mooney, Neil Wigglesworth

**Affiliations:** 1 Formally, Florence Nightingale Faculty of Nursing and Midwifery, and Palliative Care, Department of Adult Nursing, 4616 King’s College London, London, UK; 2 8945 Guy’s and St Thomas’ NHS Trust, London, UK

**Keywords:** Feasibility and acceptability, seven-day working, infection prevention and control nursing

## Abstract

**Background:**

Hospital Infection Prevention and Control Teams (IPCTs) provide clinical cover during weekdays with on call support at weekends. We report the results of a 6-month pilot of extending infection prevention and control nursing (IPCN) clinical cover to weekends at one National Health Service trust in the United Kingdom.

**Methods:**

We examined daily episodes of infection prevention and control (IPC) clinical advice given before and during the pilot of extended IPCN to weekends. Stakeholders rated the value, impact, and their awareness of the new extended IPCN cover.

**Results:**

Episodes of clinical advice given were more evenly distributed across the weeks during the pilot. Advantages for infection management, patient flow, and clinical workload were seen.

**Conclusions:**

IPCN clinical cover at weekends is feasible and valued by stakeholders.

## Introduction

The UK National Health Service (NHS) is working toward providing a 7-day clinical service as part of the Department of Health’s Sustainability and Transformation plan (King’s Fund, 2017). Seven-day clinical services in cardiac, cancer, orthopaedics, and diabetes care have been successfully introduced in some NHS Trusts (NHS Improvement, 2012; Raghavan et al., 2014).

In the UK, Infection Prevention and Control Teams (IPCTs) comprising medical and nursing personnel provide infection prevention and control cover during weekdays, with on call medical cover at weekends. Reduced infection prevention and control nursing (IPCN) cover at weekends may increase the length of stay a patient may have in isolation and increase hospital stay, adversely affecting patient flow through to discharge home. For example, patients who could be accommodated in a multi-bed bay may be held in the Emergency Department waiting for a single room, or patients may continue to occupy single rooms who no longer need to be isolated. Delays in appropriate isolation could lead to secondary and cross infection, although to date, for many organisms there is limited evidence to support this. Expanding IPCN clinical services over 7 days may help to avoid such problems (NHS services, 2013).

In August 2016, in response to the Department of Health’s plan to improve quality of care for patients and efficiency of services (NHS Improvement, 2012), IPCN services at one NHS Trust were expanded to Saturday and Sunday for a 6-month pilot. A new nursing post was created for the duration of the pilot.

The feasibility and acceptability of extending IPCN provision to weekends were not known. We conducted a feasibility and acceptability study of extending IPCN to weekends across one NHS Trust. The aims were to a) examine the feasibility and acceptability of extending IPCN cover to weekends and b) to determine the impact of weekend IPCN on work load during the week.

## Methods

### Survey

We sent a short self-completion questionnaire with six impact statements to key infection prevention and control (IPC) stakeholder groups within the Trust. Stakeholder groups comprised the Trust’s infection prevention and control team, that is, consultants, nurses, and doctors in training, bed and facility managers, cleaning services, and site nurse practitioners.

Stakeholders were asked about their awareness of the extended IPCN service to weekends, and the perceived value of the extended service. Stakeholders were also asked to score statements about a) the quality and safety of patient care resulting from the extended service; and b) flow of patients from isolation to the ward and vice versa. Scores were from 0 to 100 on a continuous scale, where 0 was the lowest score and 100 was the highest. Stakeholders were also asked to add free text comments about the weekend IPCN service.

The survey was created in SurveyMonkey (https://www.surveymonkey.com/). A link to the survey was emailed to stakeholder groups. We sent one email reminder to all groups. The survey was anonymous, and all responses were recorded in Microsoft Excel. Free text responses were examined for content. Scores were aggregated for each impact statement and presented as scores out of 100. The study was approved as a service evaluation by the Trust.

### Infection prevention and control database data

In addition to the survey, we extracted routine IPC workload data from the Trust’s IPC database (ICNet NG 1.5.7.0, Baxter International Inc. Newbury, UK). Episodes of infection prevention advice given by a member of the IPC team are recorded daily in free text with the date and time of each episode. This information is linked to patient records. Two authors (NW and LM) extracted the date, time, and number of individual episodes of clinical advice given per day before the IPCN weekend cover started, that is, from 04/01/2016 to 12/07/2016; and during the pilot, that is, from 13/07/2016 to 11/11/2016. The mean number of advice episodes per day before and during the pilot was calculated.

## Results

### Impact statement responses

Thirty stakeholders across different groups responded to the survey. Stakeholders’ awareness of the weekend service and the value of the service was scored very highly. The impact of the service on quality, safety, and patient flow also scored highly (Table 1).

**Table 1.** Stakeholder rated impact statements.

Statement	Average score
Awareness of IPCN weekend service	92/100
Value of extending IPCN to weekends	89/92
Impact of IPCN weekend service on quality and safety of patient care	88/100
Impact on patient flow and placement	85/100

### Feasibility and acceptability

Nineteen of the thirty respondents commented on the IPCN weekend service. Overall, responses were very positive. Extending IPCN cover to weekends was feasible and acceptable to stakeholders. Themes to emerge from the free text comments were: additional support for decision making, better resource management, patient movement, workflow, and understanding of IPC workload.

Stakeholders reported that patient flow from isolation had improved and that the extended IPCN provision to weekends had impacted positively on bed management especially during infection outbreaks. Stakeholders felt that clarification of diagnosis was timelier and that the availability of advice and support during weekends helped to improve the management of patients with infections as expressed by this stakeholder.

‘….Found having advice and support for infection prevention and control decision making extremely useful at weekends, some decisions/discussion would have in the past had to wait until Monday’.

IPC workflow during weekends was thought to have improved, and some stakeholders felt they had a greater understanding of the workload associated with infection prevention at weekends.

‘Huge help to the learning, understanding and workload of infection trainees at the weekend’.

There was overall agreement among stakeholders that the weekend IPCN service continue.

### Impact on workload

Data extracted from the IPC database demonstrated that during the 6 months before the pilot, episodes of infection prevention and control advice were distributed less evenly during the week (Table 2) with an average of 24 more episodes of IPC advice given on Mondays than other weekdays.

**Table 2.** Episodes of IPC advice given to stakeholders during the week and at weekends.

Weekday	Pre-pilot	Pilot
Monday	54	30
Tuesday–Friday	25	25
Saturday–Sunday	—	25

During the pilot, the mean number of episodes of advice per day was more evenly distributed across the week with an average of five more episodes of advice given on Mondays.

## Discussion

### Summary of findings

We found the introduction of weekend IPCN cover at one NHS Trust was feasible to implement and very highly valued by stakeholders. There were perceived advantages for infection management, patient flow, and bed management at weekends. The IPC workload balance over the week improved, and there was overall agreement among stakeholders that the service continue.

### Strengths of the study

Through this pilot, multidisciplinary IPC personnel and other hospital management stakeholders could consider the acceptability, value, and impact of the weekend IPCN cover on the management of patients with infections. Continuation of this service is informed by stakeholder feedback. This study has established a baseline for future IPCN service evaluations within the Trust.

### Limitations of the study

Although we gathered stakeholder’s experiences of the IPCN weekend service, patient experiences are not represented. Improvements to delayed discharge or impacts on nursing staff training and continuing professional development were not examined. These factors should be included in any future service evaluation. Furthermore, these results are limited to one acute NHS teaching hospital Trust but could be broadly applicable to other trusts with similar IPC nursing and medical team structures. For the survey, groups of stakeholders rather than individual stakeholders were emailed to protect the anonymity of Trust personnel; therefore, the survey results are presented as aggregate scores rather than separated by professional or stakeholder groups.

## Implications of the findings

During the pilot, the mean number of episodes of advice per day from Tuesday to Friday was 25; the mean number of episodes per day on Mondays was 30. The extra five episodes per day is equal to approximately 1 hour of IPCN time. This small residual ‘Monday effect’ is likely to be attributable to patterns of working, for example, the timing of microbiological specimen collection and laboratory processing and/or deferral of clinical decision making to Monday for wider consultation. It is clear from the findings that IPCN workload was more evenly distributed during the weekdays of the pilot. Therefore, the reduction in the numbers of episodes of advice given on Mondays represents a shift to giving IPC advice during weekends.

It should be noted that individual patient advice is just one aspect of the role of an IPCN and does not capture other reactive or proactive activities. Episodes of advice were recorded as an indication of workload; however, this is not a complete picture of IPC workload, for example, attending clinical meetings, ward rounds, clinical and environmental audits, meetings with estates and facilities teams, finance, and human resources are not represented.

Seven-day working has been piloted and introduced in other clinical settings in the UK (NHS Improvement, 2012; Raghavan et al., 2014), for example, in orthopaedics, cancer care – radiotherapy, older persons care, women’s services, diagnostics, diabetes care, and general medicine. As far as we are aware, 7-day IPCN services are rare in the UK NHS. Our results demonstrate this model is feasible and acceptable and offers a more balanced workload throughout the week. These results have supported the establishment of a substantive nursing post to support the 7-day IPCN initiative at the trust. Further evaluation of cost savings resulting from efficient patient flow and the extra costs of weekend work is needed. Research into delayed patient discharge from isolation is also needed.

## Conclusion

The introduction of infection prevention and control nursing at weekends is feasible, improves workload distribution over the week, and is greatly valued by stakeholders.
